# Genome-wide detection of fine-scale population stratification and long-distance dispersal of the Chinese mitten crab (*Eriocheir sinensis*)

**DOI:** 10.1080/19768354.2026.2619207

**Published:** 2026-01-31

**Authors:** Jibom Jung, Mijin Park, Donghee Kim, Hyungmin Moon, Meijun Tang, Xugan Wu, Seok Hyun Lee, Jongwoo Jung, Choongwon Jeong

**Affiliations:** a Institute for Data Innovation in Science, Seoul National University, Seoul, Republic of Korea; b School of Biological Sciences, Seoul National University, Seoul, Republic of Korea; c Key Laboratory of Exploration and Utilization of Aquatic Genetic Resources, Shanghai Ocean University, Shanghai, People’s Republic of China; d Department of Companion Animals, Silla University, Busan, Republic of Korea; e Division of EcoCreative, Ewha Womans University, Seoul, Republic of Korea; f Department of Science Education, Ewha Womans University, Seoul, Republic of Korea

**Keywords:** Decapod, East Asia, SNP genotyping, gene flow, conservation genomics

## Abstract

Understanding the population genetic structure of marine decapods is essential for their effective conservation and management, particularly for species like the Chinese mitten crab (*Eriocheir sinensis*), which exhibits a complex life cycle and high invasive potential. In this study, we applied a population genomics approach using SLAF-seq to generate genome-wide SNP data from 120 unrelated individuals collected across six locations in China and Korea. We found a fine-scale but discernible level of genetic differentiation by regional populations correlated with geography. Individuals from Seocheon (Korea) and Wenzhou (southeastern China) exclusively share a distinct genetic ancestry component that makes them divergent from the rest, which we speculate may have been introduced by hybridization with congeneric species. We detected genetic outliers (9 out of 120 individuals) that show ongoing long-distance dispersal along the coastline of the Yellow Sea, likely happening during the planktonic larval phase. Collectively, our findings provide a genomic basis for delineating management strategies, supporting informed stock enhancement, and guiding region-specific conservation efforts for *E. sinensis* across East Asia.

## Introduction

Population genetics is crucial for the conservation and management of marine decapods, which inhabit highly dynamic ecosystems. In particular, genetic diversity underpins population genetic resilience, and its reduction can elevate the risks of inbreeding depression, reduce reproductive fitness, and compromise disease resistance and adaptability (Spielman et al. 2004; Ørsted et al. 2022; Tokarskaya et al. 2024). Consequently, examining the population genetic structure of a species is essential to ensure its long-term viability. Population structure can be influenced by various factors, including environmental conditions, anthropogenic impacts, and intrinsic species traits such as larval dispersal potential (Yednock and Neigel 2011; Verry et al. 2020). While planktonic larvae can travel vast distances on ocean currents and facilitate gene flow and broad geographic distributions (Briggs et al. 2024; Gesto et al. 2024; He et al. 2024), larval dispersal may sometimes be constrained, resulting in locally differentiated populations (Cowen et al. 2006; Weersing and Toonen 2009).

The Chinese mitten crab (*Eriocheir sinensis*) is a marine decapod that exemplifies a complex life history with significant ecological and economic relevance. Uniquely, it undergoes a migratory life cycle involving freshwater habitats for growth, marine environments for reproduction, and planktonic larval stages (Anger 1991). Although native to East Asia, it has become an invasive species in Europe and North America, creating ecological and economic concerns (Hayer et al. 2019). Meanwhile, *E. sinensis* continues to serve as an important fishery resource in its native ranges in China and Korea (Zhao et al. 1998). Hence, understanding the genetic structure of *E. sinensis* is vital both for sustaining local fisheries and for mitigating its potential impacts as an invasive species.

Despite its significance, genomic-level analyses of *E. sinensis* population structure remain sparse, particularly in Korean waters. Korean populations lie at the eastern-southern margin of the species’ native range and in a region influenced by the Tsushima Warm Current, suggesting that this area may represent a biogeographic transition zone (Jung et al. 2022). Korea also supports active fisheries for *E. sinensis*, underscoring the need for population-genomic data to inform local management. Previous work using traditional genetic markers has provided valuable insights into population history in East Asia (Xu et al. 2009) and traced invasive populations in Germany to the Yangtze River (Hayer et al. 2019), but comprehensive genome-wide assessments of regional population structure remain limited. Recent findings of hybridization among *E. sinensis* and its congerners, i.e. *Eriocheir hepuensis* and *Eriocheir japonica* in China and Russia (Wang et al. 2024) further highlight the potential for complex evolutionary processes in this group. However, the extent to which these factors contribute to local population structure in Korea has not yet been explored.

In this study, we employed a population genomics approach to elucidate the genetic structure of *E. sinensis* across six locations in China and Korea. Using genome-wide single-nucleotide polymorphism (SNP) data derived from 120 individuals by SLAF-seq (Sun et al. 2013), we observe a fine-scale but noticeable genetic differentiation by their geographic location. We detect a shared genetic ancestry component between southwestern Korean (Seocheon) and southeastern Chinese (Wenzhou) populations, which may reflect introgression from congeneric species, as suggested by Wang et al. (2024). Genetic outliers prove a widespread occurrence of long-distance dispersal of *E. sinensis*, likely during the larval stage, and provide a potential explanation for limited genetic differentiation between major river systems across the Yellow Sea.

## Materials & methods

### Sample collection and DNA extraction

**Figure 1.**
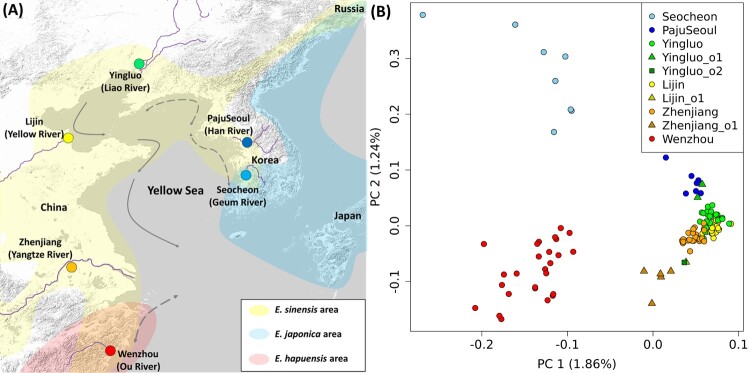
Geographic locations and genetic profiles of *Eriocheir sinensis* individuals used in this study. (a) Six location (river basins) used in this study, collected from China and Korea between 2016 and 2022. Gray arrows show ocean currents with dotted double arrows indicating seasonal changes in direction. Yellow, skyblue, and pink shades mark the distribution ranges of *E. sinensis*, *Eriocheir japonica*, and *Eriocheir hepuensis*, respectively. Purple lines indicate rivers near the sampling location. Grayshaded areas depict the basemap background (land and ocean). (b) PCA of the 120 unrelated *E. sinensis* individuals. The first two PCs were plotted on the x- and y-axis, respectively. Color-filled dots show individuals.

A total of 204 *E. sinensis* samples were collected from six locations in Korea and China between 2016 and 2022 (Figure 1(a), Table 1). Species identification was conducted through morphological observation and COI barcoding analysis. Genomic DNA was extracted using the phenol – chloroform – isoamyl alcohol method (Renshaw et al. 2015) for Korean samples, while the DNeasy Blood and Tissue Kit (Qiagen, Hilden, Germany) was employed for Chinese samples. DNA quality was assessed for SLAF-seq library preparation using a NanoDrop 2000c spectrophotometer (Thermo Fisher Scientific, Waltham, MA, USA) and by electrophoresis on a 2% agarose gel at 120 V for 15 min.

**Table 1.** Sampling details of *Eriocheir sinensis* populations collected from three Korean and four Chinese sites in 2016–2022.

Population	Collection site	Collection date	Coordinates	Number of samples
Seocheon	Geum River, Seocheon, South Korea	26 August, 2021	36.02N 126.75E	35 / 8 / 8
PajuSeoul	Han River, Seoul, South Korea	12 July, 2020	37.52N 127.10E	20 / 5 / 5
	Imjin River, Paju, South Korea	28 November, 2022	37.85N 127.76E	30 / 2 / 2
Yingluo	Liao River, Liaoning Province, China	16 October, 2016	40.70N 122.70E	30 / 26 / 23
Yingluo_o1				.. / 2 / 2
Yingluo_o2				.. / 1 / 1
Lijin	Yellow River, Shandong Province, China	16 October, 2016	37.61N 118.52E	30 / 29 / 25
Lijin_o1				.. / 1 / 1
Zhenjiang	Yangtze River, Jiangsu Province, China	26 November, 2018	32.11N 119.27E	29 / 24 / 22
Zhenjiang_o1				.. / 5 / 5
Wenzhou	Ou River, Zhejiang Province, China	25 February, 2017	28.01N 120.65E	30 / 30 / 26
**Total**	3 Korean and 4 Chinese sites			204 / 133 / 120

Notes: Numbers in the “number of samples” column represent the total number of samples collected from each site, the number of individuals used in the SLAF-seq experiment, and the number of unrelated individuals in the final data set, respectively.

### SLAF library construction

SLAF-seq is a cost-effective reduced-representation sequencing technique. Compared to RAD-seq, SLAF-seq employs a pre-designed, in silico – optimized approach that yields more predictable marker placement and improved genotyping consistency (Sun et al. 2013). The SLAF library was constructed following a previously described protocol with minor modifications by Biomarker Technologies Co. (Hong Kong). Genomic DNA from each sample was digested with the restriction enzymes HaeIII (GCCC) and Hpy166II (GTNNAC). ATP and dual-index sequencing adapters were ligated to the 3′ and 5′ ends of the digested DNA fragments, respectively. The ligated DNA was amplified by PCR, and the resulting products were purified using the E.Z.N.A. Cycle Pure Kit (Omega Bio-Tek, Norcross, GA, USA). The purified PCR products were re-digested with the same restriction enzyme, followed by ligation of ATP and Illumina sequencing adapters. The ligation products were purified using Quick Spin columns (Qiagen, Venlo, Netherlands) and separated on a 2% agarose gel. DNA fragments between 364 and 464 bp were isolated using the Gel Extraction Kit (Tiangen Biotech, Beijing, China). These SLAFs were subjected to additional PCR to incorporate barcodes. The final PCR products were re-purified and prepared for paired-end sequencing on an Illumina HiSeq platform (Illumina, San Diego, CA, USA).

### Mapping and data filtering

The SLAF-seq data of *E. sinensis* samples were aligned to the reference genome (GenBank accession ASM2467909v1; Wang et al. 2022) using the “mem” module in BWA 0.7.17 (Li et al. 2009). Individuals with mapping rates below 0.4 were excluded due to potential misclassification and failure of DNA extraction. Reads with a Phred-scaled mapping quality score below 30 were filtered out using SAMtools 1.19.2 (Li et al. 2009). Bacterial contamination was assessed using Kraken 2.1.3 (Wood et al. 2019) with the minikraken2_v1_8GB database.

### Variant calling and genotyping

Variant calling was performed using the HaplotypeCaller module in GATK 4.1.2.0, and gVCF files were combined using the CombineGVCFs module (Poplin et al. 2017). Variants were called using the GenotypeGVCFs module, and multi-allelic and low-quality SNPs were filtered using the VariantFiltration module with the criteria “DP < 25.0 || DP > 650.0 || QD < 2.0 || SOR > 3.0 || FS > 60.0 || MQ < =  40.0 || MQRankSum < −12.5 || ReadPosRankSum < −8.0 || ExcessHet > 10.0”. Due to the low per-site coverage, diploid genotype calling would be unreliable; therefore, random pseudo-haploid genotypes were generated by selecting one high-quality base (Phred score ≥ 30) using pileupCaller 1.5.2 with the “randomHaploid” option (https://github.com/stschiff/sequenceTools; accessed 29 Nov 2024). Pairwise mismatch rates (PMR) were calculated as the proportion of SNP sites with valid genotypes in both individuals at which their random haploid genotypes differ. The PMR value of an individual pair with their coefficient of relationship = r is expected to be times of that of an unrelated pair of individuals (Kennett et al. 2017). For example, genotypes from two independently built libraries of an individual, or genotypes of identical twins (i.e. r = 1), are expected to have the PMR value that is a half of PMR between unrelated individuals. PMRs were used to detect pairs of nearly identical individuals, from which one with lower coverage was removed. Final SNP filtering was conducted in PLINK (Chang et al. 2015) using the parameters “–maf 0.01 and –geno 0.5”, corresponding to remove SNPs missing in >50% individuals or SNPs with minor allele frequency lower than 0.01. Finally, a set of 193,825 polymorphic SNPs were retained for downstream analysis.

### Principal Component Analysis, F_ST_, and ADMIXTURE

To investigate the genomic structure of *E. sinensis*, we conducted PCA (Price et al. 2006) and ADMIXTURE (Alexander et al. 2009). To address genotype missingness, we filtered SNPs with >60% missing data (n = 5) and individuals with >70% missing data (n = 9). Then, we removed rare SNPs with minor allele counts 2 or less, resulting in the final data set of 127,841 SNPs and 124 individuals. The genotype matrix was centralized by subtracting the mean allele frequency, and a genetic similarity matrix was constructed using the mean product of centralized genotype values for complete observation pairs. PCA was performed via eigen decomposition of the similarity matrix using the eigen() function in R v4.2.1 with default parameters, and the first two components were used to visualize genetic structure. We applied the eigen() function to two subsets of the 124 × 124 similarity matrix: 120 × 120 matrix after removing four potentially duplicate individuals, 86 × 86 matrix after further removing individuals from the Seocheon and Wenzhou. ADMIXTURE analyses were conducted for K = 2–6 using a data set including 120 unrelated individuals and 127,841 SNPs. Five-fold cross-validation errors were calculated to identify the optimal K, and population structure was visualized using R, generating stacked bar plots for K values from 2 to 6 for the run with the highest log likelihood for each K value. *F_ST_* was calculated for all 15 pairs of six main groups (Lijin, PajuSeoul, Seocheon, Wenzhou, Yingluo, Zhenjiang), for all six groups together, and for four groups excluding Seocheon and Wenzhou, respectively, using the same data set. Weir and Cockerham estimator of *F_ST_* (Weir and Cockerham 1984) was used. To account for low coverage and uneven sample size, empirical *p*-value of the *F_ST_* values were estimated following a permutation approach. Specifically, group labels were randomly reassigned across individuals belonging to the six main groups and *F_ST_* was calculated for the group label-permuted data set. After repeating this process 2,500 times, the empirical *p*-value was calculated for each *F_ST_* value as follows: ((the number of permuted data set with *F_ST_* ≥ observed *F_ST_*) + 1) / 2,501.

### f-statistics

We performed *f*-statistics analysis using the R packages admixtools 2.0.6 (https://github.com/uqrmaie1/admixtools) and gtools 3.9.5 (https://github.com/cran/gtools). The outgroup *f₃* statistic of the form *f₃*(outgroup; A, B) measures the covariance of the allele frequency difference between (outgroup, A) and (outgroup, B) pairs across genome-wide SNPs, therefore quantifies the amount of shared genetic drift between populations A and B since their divergence from an outgroup (Reich et al. 2009; Patterson et al. 2012). Overall, larger outgroup *f_3_* statistics between populations A and B suggest closer genetic relationship between them. The *f₄* statistic of the form *f₄*(A, B; C, D) measures the covariance of the allele frequency difference between (A, B) and (C, D) pairs across genome-wide SNPs (Green et al. 2010; Patterson et al. 2012). When A is an outgroup to the others (therefore involving no gene flow) and B is overall more distant from C and D than they are to each other, statistically significant deviation from zero indicates an asymmetric relationship of C and D with regard to B. More specifically, significant positive *f_4_* statistics indicate a gene flow between B and D, while significant negative statistics indicate a gene flow between B and C (Byeon and Jeong 2024). We calculated *f₃* statistics for all triplets of populations using the qp3pop function (Table S1), and *f₄* statistics for all quartets of populations using the qpdstat function as implemented in the admixtools 2.0.6 package in R (Table S2).

## Results

### Geographic population stratification of *E. sinensis* revealed by genome-wide data

**Figure 2.**
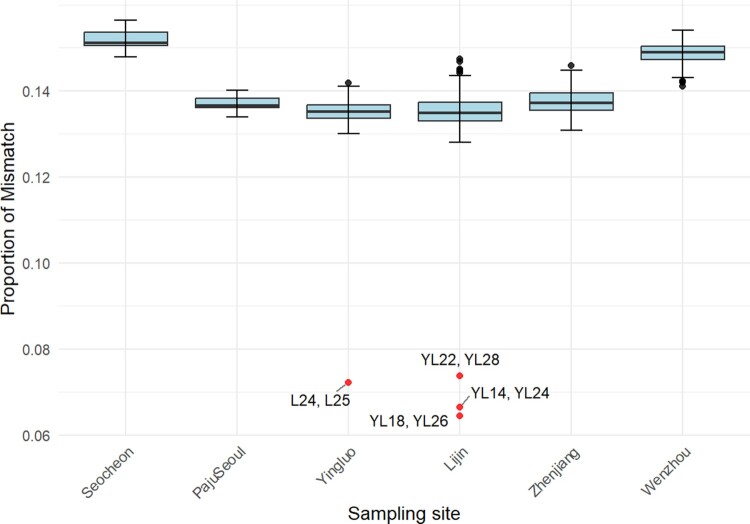
Pairwise genotype mismatch rate (PMR) of *Eriocheir sinensis* individual pairs within each of the six locations. In each box-whisker plot, the box mark the median and the interquartile range, the whisker mark ±1.5×(interquartile range), and circles mark the outliers beyond the range of the whisker. Red dots represent four pairs of duplicate individuals, whose PMR values are around a half of the population median.

COI barcoding verified that all 204 individuals in this study belonged to *E. sinensis*, as their sequences grouped within the main *E. sinensis* clade represented in the The Barcode of Life Data Systems (BOLD) reference database. A total of 204 *E. sinensis* individuals were initially subjected to SLAF-seq. Although DNA was successfully extracted from all individuals, 71 samples were excluded from downstream analysis due to insufficient sequencing depth, poor read quality, or low mapping rates. The remaining 133 samples passed initial quality checks (Table 1), and after removing 9 additional low-quality individuals with low mapping rates or SNP coverage, 124 individuals were retained for downstream analyses (Table S3). The total read count ranged from 2,481,368–29,130,138 (mean: 8,045,162 reads). The mapping rate ranged from 50.4% to 91.4% (mean: 86.5%), and the proportion of high-quality reads (Phread-scaled mapping quality score 30 or higher) varied between 53.3% and 66.5% (mean: 61.1%). The average GC content was 42.8%, consistent with expectations for this species. To mitigate low coverage inherent to SLAF-seq data, we constructed haploid genotypes by selecting a single high-quality base per individual at each position. We further filtered for polymorphic bi-allelic SNPs containing fewer than 50% missing genotypes, yielding 193,825 high-confidence bi-allelic SNPs. Individual SNP coverage ranged from 67,062–189,332 across 124 individuals. Finally, four pairs of supposedly identical individuals were identified using PMR (Figure 2, Table S4). They may represent true identical twins, samples from a highly inbred subpopulation, or sample/DNA labeling mistake, however, the current dataset does not provide sufficient resolution to distinguish among these possibilities. To remove bias in the downstream population genetic analysis due to the inclusion of close genetic relatives, one with lower SNP coverage per pair was removed, resulting in 120 unrelated individuals in the final data set (Tables 1 and S3).

PCA revealed an overall correspondence between genomic variation and geographic origin (Figure 1(b), Table 1). PC1 separated individuals from Seocheon (n = 8) and Wenzhou (n = 26) from the others, and PC2 separated Seocheon and Wenzhou individuals. Individuals from the other four locations were placed close to each other on the PC space but tended to be separated by locations (Figures 1(b) and S1). Interestingly, their relative position on the PC space seemed to be correlated with their geographic location (Tables 1 and S3). First, PajuSeoul in Korea (n = 7) were displaced from the Chinese individuals toward Seocheon, a more southern Korean location. Second, most Yingluo individuals (n = 24) from the Liao River in northeast China were slightly displaced from the other Chinese individuals toward the Korean individuals upwardly along PC2 (Figure 1(b)). Two Yingluo individuals were markedly separated from the others upwardly along PC2 and overlapped with PajuSeoul: we grouped them separately for the group-based analyses (hereafter “Yingluo_o1”). On the other hand, another Yingluo individual was displaced toward Wenzhou and accordingly grouped separately (hereafter “Yingluo_o2”). Third, Zhenjiang individuals were displaced from the other Chinese ones toward Wenzhou, with 5 out of 27 individuals being displaced further toward Wenzhou: we grouped them separately as Zhenjizng (n = 22) and Zhenjiang_o1 (n = 5). Last, one out of 26 Lijin individuals was separated from the others and overlapped with Yingluo_o2: it was grouped separated (hereafter “Lijin_o1”) (Table S5).

**Figure 3.**
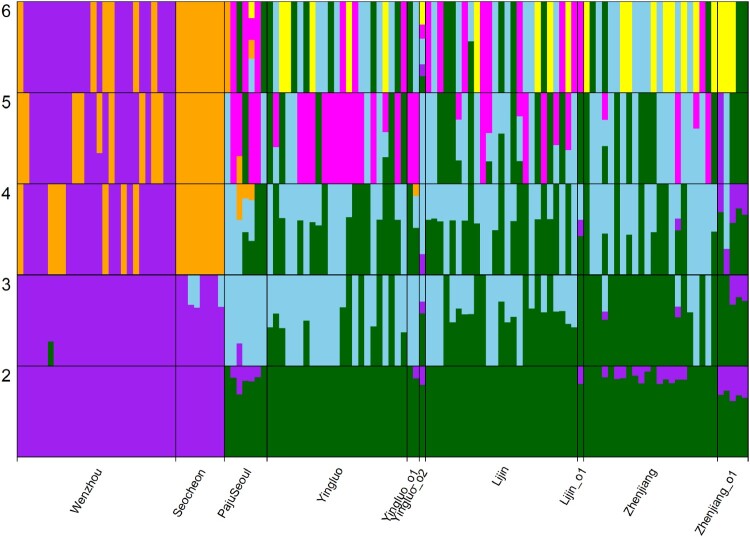
Ancestry proportions of *Eriocheir sinensis* individuals inferred from ADMIXTURE analysis at K = 2 to K = 6. Each vertical bar represents one individual, and colors indicate the proportion of inferred ancestry from each genetic cluster. Individuals are grouped by sampling locality: Wenzhou, Seocheon, PajuSeoul, Yingluo, Yingluo_01, Yingluo_02, Lijin, Lijin_01, Zhenjiang, and Zhenjiang_01.

**Figure 4.**
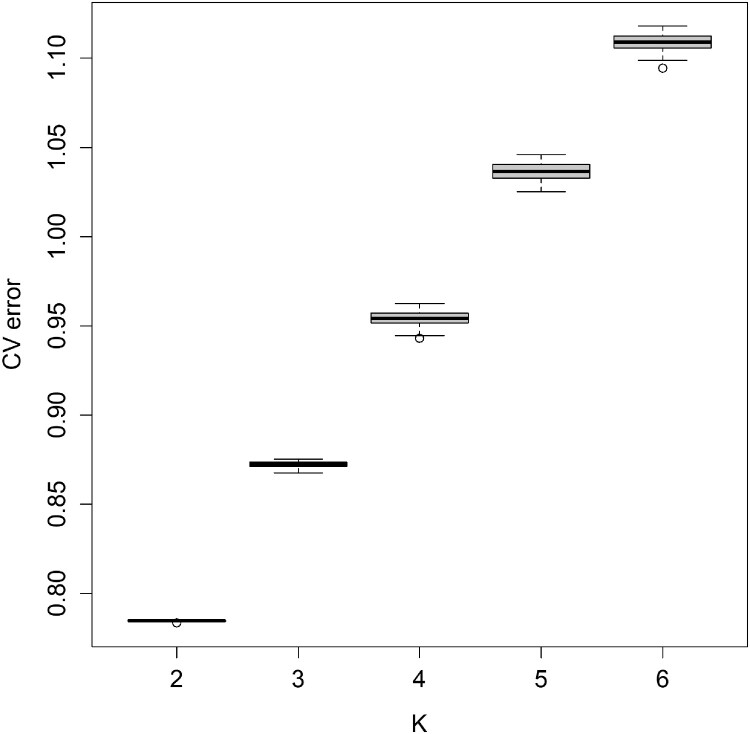
Cross-validation (CV) errors from ADMIXTURE analysis of *Eriocheir sinensis* for K = 2 to K = 6. Boxplots show the distribution of CV errors across 20 replicates for each K value. The lowest CV error was observed at K = 2, suggesting it as the most likely number of ancestral populations. These results are consistent with pairwise *F_ST_* patterns among populations.

Unsupervised clustering results from ADMIXTURE were broadly consistent with the PCA results (Figure 3). The lowest CV error at K = 2, together with the instability of cross-validation and log-likelihood estimates at higher K values, supported a two-cluster model as the most parsimonious representation. At K = 2, where the smallest CV error was observed, Seocheon and Wenzhou formed one cluster while the remaining four populations grouped into a second cluster, mirroring the structure observedalong PC1 (Figure 4, Table 2).

**Table 2.** ADMIXTURE analysis results for *Eriocheir sinensis* populations across K = 2 to K = 6.

	K=2	K=3	K=4	K=5	K=6
LogLikelihood	−6799845.563	−6655032.994	−6501140.113	−6369669.293	−6247744.443
	−6799873.895	−6650869.67	−6516188.954	−6381399.669	−6255130.672
	−6799840.403	−6649656.286	−6509283.18	−6365747.726	−6270086.883
	−6799840.403	−6653504.723	−6509089.726	−6368644.866	−6240943.325
	−6799840.403	−6655330.576	−6506742.507	−6375457.158	−6240150.431
	−6799840.403	−6653187.183	−6518107.902	−6407081.063	−6255962.533
	−6799840.403	−6652223.832	−6509283.18	−6389482.921	−6253624.375
	−6799840.403	−6654663.521	−6522732.451	−6384435.4	−6254713.071
	−6799840.403	−6653709.126	−6509089.726	−6363549.196	−6241738.024
	−6799840.403	−6667445.172	−6515283.893	−6384907.469	−6256857.331
	−6799840.403	−6654663.521	−6519206.224	−6370040.023	−6231973.616
	−6799840.403	−6647147.673	−6515467.348	−6380495.497	−6256438.342
	−6799840.403	−6667445.172	−6521491.829	−6367028.678	−6237442.078
	−6799871.042	−6653432.61	−6500261.125	−6365271.376	−6239216.802
	−6799871.042	−6651445.797	−6506742.507	−6388691.304	−6252309.198
	−6799871.042	−6648025.511	−6501495.991	−6391954.419	−6250050.429
	−6799871.042	−6651445.797	−6506190.398	−6375177.56	−6248204.485
	−6799871.042	−6657483.358	−6518819.745	−6374876.864	−6266086.887
	−6799871.042	−6652292.711	−6510294.654	−6381884.579	−6220038.266
	−6799871.042	−6651586.192	−6523429.029	−6360857.715	−6243697.429
Cverror	0.78338	0.87221	0.95636	1.03839	1.11097
	0.78446	0.86747	0.96092	1.03599	1.11041
	0.78513	0.87309	0.95245	1.02885	1.11584
	0.78513	0.87477	0.94302	1.04026	1.11597
	0.78513	0.87269	0.95367	1.03624	1.09439
	0.78440	0.87184	0.95621	1.04178	1.10626
	0.78440	0.87521	0.95245	1.04107	1.09996
	0.78440	0.87459	0.96050	1.04087	1.10846
	0.78440	0.87172	0.94302	1.03572	1.11194
	0.78440	0.87356	0.95671	1.04596	1.11296
	0.78440	0.87459	0.96239	1.03763	1.10324
	0.78440	0.87004	0.95735	1.03223	1.11117
	0.78440	0.87356	0.95828	1.02528	1.11534
	0.78490	0.87106	0.94453	1.03661	1.10879
	0.78490	0.87103	0.95367	1.04110	1.10918
	0.78490	0.86942	0.95603	1.03328	1.10765
	0.78490	0.87103	0.95098	1.02934	1.09886
	0.78490	0.87236	0.95457	1.02673	1.11797
	0.78490	0.87173	0.95323	1.04028	1.10565
	0.78490	0.87271	0.94703	1.03616	1.10594

Notes: Log-likelihood values from 20 independent runs for each K value with the highest log-likelihood for each K was used for visualizing ancestry proportions. Cross-validation (CV) errors for each K value, used to identify the optimal K based on the lowest CV error was lowest at K = 2, gradually increasing with higher K values, suggesting that K = 2 is the most likely number of ancestral populations in the dataset.

Likewise, our *F_ST_* analysis showed overall consistent results with PCA and ADMIXTURE while the nominal values of *F_ST_* were sensitive to small sample size (Table 3). *F_ST_* across all six main groups (Lijin, PajuSeoul, Seocheon, Wenzhou, Yingluo, and Zhenjiang) was relatively small but statistically significant (0.060; permutation-based empirical *p*-value = 4.00 × 10^−4^; Table 3), consistent with the small amounts of total variation explained by the top PCs. *F_ST_* of four populations excluding Seocheon and Wenzhou became smaller but still statistically significant (0.046; empirical *p*-value = 4.00 × 10^−4^; Table 3). When calculating *F_ST_* in a pair-wise manner, both Seocheon and Wenzhou were significantly differentiated from other Chinese populations (Lijin, Yingluo, and Zhenjiang; empirical *p*-value≤8.00 × 10^−4^; Table 3), while Seocheon and Wenzhou were less differentiated (empirical *p*-value = 3.68 × 10^−2^; Table 3). Nominally higher pairwise *F_ST_* values involving PajuSeoul or Seocheon likely reflect greater statistical uncertainty due to their smaller sample sizes. For example, the largest *F_ST_* was observed between these two populations (0.123), but this value is not statistically significant (empirical *p* = 0.231; Table 3), indicating that sampling variance rather than true divergence might account for the observed estimate.

**Table 3.** Pairwise genetic differentiation (*F_ST_*) and empirical *p-*values among six populations of *Eriocheir sinensis* based on SLAF-seq SNP data.

pair1	pair2	*F_ST_*	Empirical *p-*value
Lijin	PajuSeoul	0.07704	2.04E-01
Lijin	Seocheon	0.09548	4.00E-04
Lijin	Wenzhou	0.05731	4.00E-04
Lijin	Yingluo	0.03340	7.36E-02
Lijin	Zhenjiang	0.03766	2.40E-03
PajuSeoul	Seocheon	0.12319	2.31E-01
PajuSeoul	Wenzhou	0.08780	2.20E-02
PajuSeoul	Yingluo	0.07682	2.94E-01
PajuSeoul	Zhenjiang	0.08424	8.80E-02
Seocheon	Wenzhou	0.07412	3.68E-02
Seocheon	Yingluo	0.09511	8.00E-04
Seocheon	Zhenjiang	0.09666	4.00E-04
Wenzhou	Yingluo	0.05901	4.00E-04
Wenzhou	Zhenjiang	0.05440	4.00E-04
Yingluo	Zhenjiang	0.04147	4.00E-04
Multi*F_ST_*	all	0.06019	4.00E-04
Multi*F_ST_*	subset	0.04636	4.00E-04

Notes: *F_ST_* values were calculated using the Weir and Cockerham (1984) estimator. Empirical *p-*values were obtained via 2,500 permutations by randomly reassigning group labels. The six populations include Lijin, PajuSeoul, Seocheon, Wenzhou, Yingluo, and Zhenjiang.

### A distinct genetic component shared by Seocheon and Wenzhou populations

**Figure 5.**
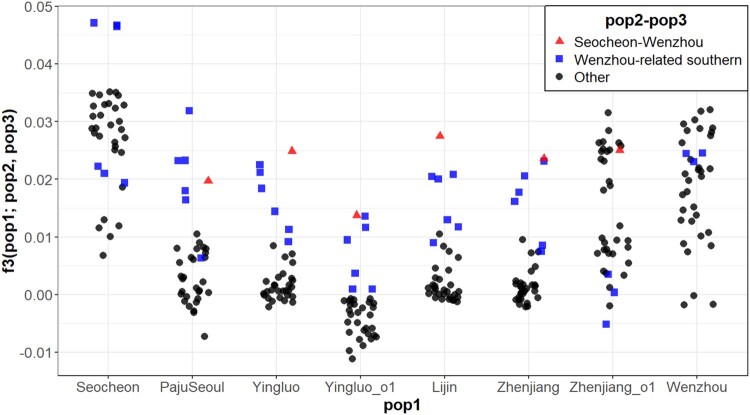
Outgroup *f_3_* statistics for *Eriocheir sinensis* across different populations. For each population (pop 1), we show *f_3_* statistics of the form *f_3_*(pop1; pop2, pop3) for all pairs of pops 2 and 3. The Seocheon and Wenzhou pair (red triangles) consistently shows high value suggesting a shared genetic drift exclusively between them. Outlier groups with Wenzhou-related southern genetic profiles (Yingluo_o2, Lijin_o1, and Zhenjiang_o1) also show high outgroup *f_3_* values between them as well as with Wenzhou (blue squares). The populations Yinluo_o2 and Lijin_o1 were excluded from the analysis due to the absence of valid data (all values were missing).

While PCA results showed distinct genetic profiles of Seocheon and Wenzhou compared to the other sites, multiple different hypotheses might have produced the observed pattern. One hypothesis was that Seocheon and Wenzhou became distinct simply due to extreme genetic drift that happened separately in those two. An alternative was that they exclusively share a distinct genetic component despite a large geographic distance. If the former hypothesis was held, one can expect no shared genetic drift between Seocheon and Wenzhou. In contrast, if the latter held, one can expect a substantial amount of genetic drift shared between them. Outgroup *f_3_* statistics of the form *f_3_*(outgroup; Seocheon, Wenzhou), using the other four major groups as “outgroup”, consistently showed higher values (0.2390) than the *f_3_* values of the other pairs, i.e. *f_3_*(outgroup; other 1, other 2) (0.047), suggesting that Seocheon and Wenzhou indeed shared a much higher amount of genetic drift than the others (Figure 5, Table S1).

**Figure 6.**
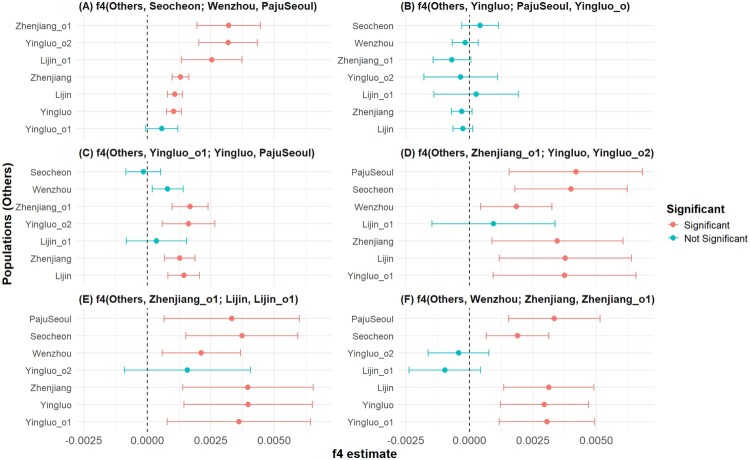
*f_4_* test results of selected *Eriocheir sinensis* populations. We present the following combinations of *f_4_* statistics. (a) *f4*(Others, PajuSeoul; Wenzhou, Seocheon); (b) *f_4_*(Others, Yingluo; PajuSeoul, Yingluo_o); (c) *f_4_*(Others, Yingluo_o1; Yingluo, PajuSeoul); (d) *f_4_*(Others, Zhenjiang_o1; Yingluo, Yingluo_o2); (e) *f_4_*(Others, Zhenjiang_o1; Lijin, Lijin_o1); (f) *f_4_*(Others, Wenzhou; Zhenjiang, Zhenjiang_o1). The horizontal lines show ±2 standard error measures (s.e.m.) estimated by 3,000,000 bp block jackknifing. Blue and red points indicate *f_4_* statistics deviating ≥2 and <2 s.e.m. from zero, respectively. The dashed line represents an *f_4_* estimate of zero.

However, as their distinct position on PC2 and large geographic distance suggested, Seocheon and Wenzhou do not form a clade against the other groups. Most notably, Seocheon was significantly closer to the other Korean population, PajuSeoul, compared to Wenzhou as measured by *f_4_* statistics of form *f_4_*(Seocheon, Wenzhou; PajuSeoul, others) > 4.1 standard error measures (s.e.m.) for 6 out of 7 other groups (Figure 6, Tables 4 and S2). The only exception was Yingluo_o1, an outlier group overlapping with PajuSeoul, that showed no significant statistic.

**Table 4.** Key *f_4_* test results among *Eriocheir sinensis* populations.

Pop1	Pop2	Pop3	Pop4	*f_4_*	s.e.m.	Z	nSNPs
Lijin	PajuSeoul	Wenzhou	Seocheon	0.00109	0.00015	7.03	51884
Lijin_o1	PajuSeoul	Wenzhou	Seocheon	0.00254	0.00061	4.146	25396
Yingluo	PajuSeoul	Wenzhou	Seocheon	0.00104	0.00015	7.039	51884
Yingluo_o1	PajuSeoul	Wenzhou	Seocheon	0.00057	0.00032	1.771	47520
Yingluo_o2	PajuSeoul	Wenzhou	Seocheon	0.00319	0.00059	5.361	36566
Zhenjiang	PajuSeoul	Wenzhou	Seocheon	0.00131	0.00017	7.524	51884
Zhenjiang_o1	PajuSeoul	Wenzhou	Seocheon	0.00321	0.00064	5.039	51733
Lijin	Yingluo	PajuSeoul	Yingluo_o1	−0.00026	0.0002	−1.312	47636
Lijin_o1	Yingluo	PajuSeoul	Yingluo_o1	0.00027	0.00085	0.318	23351
Seocheon	Yingluo	PajuSeoul	Yingluo_o1	0.00042	0.00037	1.135	47520
Wenzhou	Yingluo	PajuSeoul	Yingluo_o1	−0.00017	0.00026	−0.631	47636
Yingluo_o2	Yingluo	PajuSeoul	Yingluo_o1	−0.00034	0.00074	−0.465	33834
Zhenjiang	Yingluo	PajuSeoul	Yingluo_o1	−0.0003	0.00021	−1.448	47636
Zhenjiang_o1	Yingluo	PajuSeoul	Yingluo_o1	−0.00069	0.00038	−1.791	47506
Lijin	Yingluo_o1	Yingluo	PajuSeoul	0.00144	0.00032	4.512	47636
Lijin_o1	Yingluo_o1	Yingluo	PajuSeoul	0.00036	0.00061	0.598	23351
Seocheon	Yingluo_o1	Yingluo	PajuSeoul	−0.00016	0.00035	−0.461	47520
Wenzhou	Yingluo_o1	Yingluo	PajuSeoul	0.0008	0.00031	2.572	47636
Yingluo_o2	Yingluo_o1	Yingluo	PajuSeoul	0.00162	0.00053	3.062	33834
Zhenjiang	Yingluo_o1	Yingluo	PajuSeoul	0.00128	0.00031	4.143	47636
Zhenjiang_o1	Yingluo_o1	Yingluo	PajuSeoul	0.00169	0.00036	4.717	47506
Lijin	Zhenjiang_o1	Yingluo	Yingluo_o2	0.00378	0.00133	2.835	36743
Lijin_o1	Zhenjiang_o1	Yingluo	Yingluo_o2	0.00095	0.00124	0.77	18455
PajuSeoul	Zhenjiang_o1	Yingluo	Yingluo_o2	0.0042	0.00134	3.128	36586
Seocheon	Zhenjiang_o1	Yingluo	Yingluo_o2	0.00401	0.00113	3.545	36611
Wenzhou	Zhenjiang_o1	Yingluo	Yingluo_o2	0.00185	0.00072	2.557	36743
Yingluo_o1	Zhenjiang_o1	Yingluo	Yingluo_o2	0.00375	0.00144	2.598	33894
Zhenjiang	Zhenjiang_o1	Yingluo	Yingluo_o2	0.00347	0.00132	2.632	36743
PajuSeoul	Zhenjiang_o1	Lijin	Lijin_o1	0.00333	0.00136	2.439	25441
Seocheon	Zhenjiang_o1	Lijin	Lijin_o1	0.00373	0.00113	3.293	25463
Wenzhou	Zhenjiang_o1	Lijin	Lijin_o1	0.00213	0.00079	2.684	25570
Yingluo	Zhenjiang_o1	Lijin	Lijin_o1	0.00398	0.00129	3.088	25570
Yingluo_o1	Zhenjiang_o1	Lijin	Lijin_o1	0.00361	0.00144	2.504	23422
Yingluo_o2	Zhenjiang_o1	Lijin	Lijin_o1	0.00158	0.00127	1.245	18455
Zhenjiang	Zhenjiang_o1	Lijin	Lijin_o1	0.00397	0.00131	3.022	25570
Lijin	Wenzhou	Zhenjiang	Zhenjiang_o1	0.00314	0.00091	3.459	52035
Lijin_o1	Wenzhou	Zhenjiang	Zhenjiang_o1	−0.00097	0.00072	−1.348	25570
PajuSeoul	Wenzhou	Zhenjiang	Zhenjiang_o1	0.00335	0.00092	3.65	51862
Seocheon	Wenzhou	Zhenjiang	Zhenjiang_o1	0.0019	0.00063	3.027	51883
Yingluo	Wenzhou	Zhenjiang	Zhenjiang_o1	0.00296	0.00089	3.329	52035
Yingluo_o1	Wenzhou	Zhenjiang	Zhenjiang_o1	0.00306	0.00096	3.187	47661
Yingluo_o2	Wenzhou	Zhenjiang	Zhenjiang_o1	−0.00043	0.00061	−0.698	36743

Notes: We show *f_4_* results presented in Figure 4: Selected populations are as follows: *f_4_*(Others, PajuSeoul; Wenzhou, Seocheon); *f_4_*(Others, Yingluo; PajuSeoul, Yingluo_o1); *f_4_*(Others, Yingluo_o1; Yingluo, PajuSeoul); *f_4_*(Others, Zhenjiang_o1; Yingluo, Yingluo_o2); *f_4_*(Others, Zhenjiang_o1; Lijin, Lijin_o1); and *f_4_*(Others, Wenzhou; Zhenjiang, Zhenjiang_o1). S.e.m. are calculated by 3,000,000 bp block jackknifing as implemented in the *f_4_* function from admixtools2.

### Long-distance dispersal of *E. sinensis* shown by genetic outliers

While the genome-wide data of *E. sinensis* individuals showed a geographic population stratification, it should be noted that the degree of genetic stratification remained minute. Genome-wide differentiation across regions was limited, as reflected in low pairwise *F_ST_* values (generally 0.03–0.06) and the lack of statistical support for the highest estimate (PajuSeoul – Seocheon: *F_ST_* = 0.123, *p* = 0.231; Table 3). Excluding somewhat distinct Seocheon and Wenzhou populations, these values were similarly low: 1.42% and 1.33% respectively (Figure S1). We detected genetic outliers that may suggest recurrent mixing between different *E. sinensis* populations, either by natural or human-mediated means. First, two Yingluo_o1 individuals were indistinguishable from PajuSeoul but distinct from Yingluo, showing an ongoing gene flow from Korea to northeast China across the Yellow Sea: |*f_4_*(Others, Yingluo; PajuSeoul, Yingluo_o1)| < 1.8 s.e.m. for all 7 other groups but *f_4_*(Others, Yingluo_o1; Yingluo, PajuSeoul) > 3 s.e.m. for 4 of 7 other groups (Table 4). Second, the Yingluo_o2 and Lijin_o1 outliers are closer to southern Chinese populations than Yingluo/Lijin are, suggesting a gene flow from a southern source into the Liao and Yellow River regions: *f_4_*(Others, Zhenjiang_o1; Yingluo, Yingluo_o2) > 2.5 s.e.m. and *f_4_*(Others, Zhenjiang_o1; Lijin, Lijin_o1) > 2.4 s.e.m. for 6 of 7 other groups (Table 4). Third, the Zhenjiang_o1 outliers were likewise closer to Wenzhou than Zhenjiang: *f_4_*(Others, Wenzhou; Zhenjiang, Zhenjiang_o1) > 3.0 s.e.m. for 5 of 7 other groups (Table 4).

## Discussion

The overall small but detectable level of population differentiation detected in *E. sinensis* suggests substantial gene flow among river systems despite their long-term geographic isolation from the end of the Last Glacial Maximum. Similar fine-scale geographic patterns have been reported in some marine decapod (Dang et al. 2019), although they are not universally observed across taxa (Al-Breiki et al. 2018; Briggs et al. 2024; Gesto et al. 2024; He et al. 2024). Because adult Chinese mitten crabs live in fresh water, they would have had no opportunity to migrate among the six major rivers since the post-Last Glacial Maximum rise in sea level that disconnected these systems (Yoo et al. 2016). Therefore, the very low degree of population stratification we observe provides compelling evidence that gene flow has been maintained primarily through long-distance dispersal of planktonic larvae. Critically, this interpretation is strongly supported by the species’ larval biology: *E. sinensis* remains in the planktonic phase for 0.5–3 months (typically ∼2 months), allowing a theoretical dispersal distance of up to 600 km (Tilburg et al. 2011). Still, larval dispersal is seasonal, with survival optimized near 18°C and 25‰ salinity (Anger 1991), conditions typically encountered estuarine during June – August (Choi et al. 1999; Qin et al. 2023).

**Figure 7.**
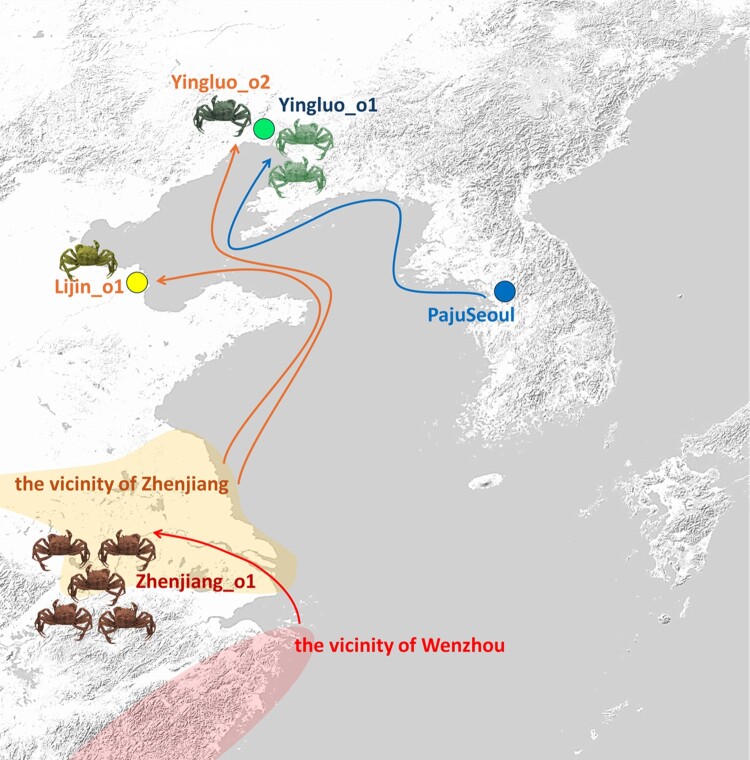
Hypothetical origins and dispersal routes of *Eriocheir sinensis* genetic outliers. Colored arrows indicate the proposed dispersal pathways based on genetic and geographical data. Crab icons denote the individual number of outlier populations, and colors of these icons represent the current affiliation of outlier populations in Figure 1. The color of allows, colored circles, and colored area indicate the origin of outlier populations noted in Figure 1.

Our identification of genetic outliers indeed corroborates this explanation (Figure 7). First, two Yingluo_o1 individuals are likely to originate from the Han and Imjin River region in Korea, ca. 900 km away. Second, Yingluo_o2 and Lijin_o1 outliers show a genetic profile close to Zhenjiang, placed on the PC space between Zhenjiang and Zhenjiang_o1, therefore suggest migrations from the Yangtze River region to as north as the Liao River. Third, the Zhenjiang_o1 individuals are closer to Wenzhou than the main Zhenjiang individuals do, suggesting a migration and mixture from the Ou River region or a migration from an unknown source between the Yangtze and Ou River regions.

It is noteworthy that all the detected outliers (n = 9 out of 120 observed individuals; 7.5%) implied a south-to-north migration: i.e. they genetically resemble more southern sources than their location of capture. Long-distance larval dispersal has long been proposed as a driver of weak population structure in small marine invertebrates, and our identification of genetic outliers in *E. sinensis* provided direct evidence for such long-distance movement. In particular, Yingluo_o2 and Lijin_o1 appeared closely related to Zhenjiang_o1, suggesting that these outliers scattered along the eastern Chinese coast might have shared a common southern origin. Comparable northward range connectivity across presumed geographic barriers has been reported for amphibians such as *Rana coreana* across the Changbai Mountain Range (Borzée et al. 2025).

Spring coastal currents near Yingluo and Lijin generally flow southward during the larval emergence period (Figure 1(a)), thus this cannot explain the observed south-to-north genetic signal. However, seasonal current reversals and mesoscale eddy activity have been reported for the Yellow Sea and East China Sea later in the year (e.g. Naimie et al. 2001; Lie and Cho 2016) and they may allow juvenile-stage transport from south to north. Additionally, human-mediated activities, such as transport via shipping or aquaculture, may have also contributed to the south-to-north dispersal, as exemplified by a well-documented case of human-mediated dispersal in invaded European waters (e.g. Tilburg et al. 2011; Hayer et al. 2019). While larval dispersal remains a plausible mechanism for the presence of genetic outliers, we also acknowledge that human-mediated processes, such as aquaculture introductions, commercial stocking, or accidental transport via trade, may have played a significant role. Notably, *E. sinensis*, although native to East Asia, was first recorded in the Aller River in Germany in 1912, a case widely attributed to accidental introduction via ballast water from trade vessels operating in the late 19th to early twentieth century (Hayer et al. 2019). The species subsequently spread across northern Europe, likely facilitated by continued maritime shipping and interconnected river systems. Given that only a small fraction of individuals (7.3%) showed outlier genotypes while the majority retained clear geographic clustering, natural processes likely represent the primary driver of population structure in our dataset in the native habitat, with human-mediated movement contributing only secondary, localized effects. Similar patterns of human-mediated aquatic species dispersal have been documented for invasive amphibians in Korea (Andersen et al. 2025). Future studies incorporating aquaculture records and more extensive geographic sampling will be essential to disentangle the relative contributions of natural and human-mediated dispersal.

Despite the overall low level of divergence between regional populations, the strongest divergence was found between Seocheon/Wenzhou and others (Figure 1(b)). Interestingly, the distinct genetic profiles of Seocheon and Wenzhou seem to be due to their sharing of a divergent genetic component not found in the other populations (Figure 5). Contemporary direct gene flow between the two regions seems unlikely given the long geographic distance over the Yellow Sea, although historical movement or introgression cannot be ruled out. In addition, the genetic outliers we identified reflect recent, individual-level dispersal events, whereas the shared divergent component in Seocheon and Wenzhou most likely represents a much older, historical introgression event involving congeneric species. Thus, the temporal scale of movement differs substantially between these phenomena. We speculate that hybridization with congeneric species, *E. japonica* and/or *E. hepuensis*, may be the source of the divergent genetic component in Seocheon and Wenzhou. Mitochondrial studies have documented the coexistence of *E. sinensis* and *E. japonica* in the Geum River basin near Seocheon (Jung et al. 2022), and additional evidence from Xu et al. (2009) and Clark et al. (2023) indicates admixture with *E. hepuensis* in southern China near Wenzhou and with *E, japonica* in the Russian Far East. This interpretation is strongly supported by Wang et al. (2024), who provided strong genome-wide evidence for introgression among *E. sinensis*, *E. japonica*, and *E. hepuensis* in southern China. Future genomic studies including these congeneric species will allow us to test this hybridization hypothesis, as well as tracking introgressed genes in the hybridizing *E. sinensis* populations in Seocheon and Wenzhou.

Finally, the lack of strong population divergence provides useful guidance for conservation genetic strategies in *E. sinensis*. The introduction of individuals from different river regions whose genetic backgrounds are broadly similar may be considered a relatively safe option for restoring depleted local populations. In practice, source populations should be selected from genetically close river systems, preferably those with low pairwise *F_ST_* values and no evidence of recent introgression of congenerics. Particular caution will be required in the Geum River and Ou River regions, where potential ongoing hybridization events may complicate translocation decisions. Furthermore, any supplementation program should include routine genetic monitoring using genome-wide SNP markers to evaluate allelic diversity, inbreeding levels, and ancestry composition to ensure that translocations do not inadvertently alter local genetic structure.

Considering the economic importance of *E. sinensis* as a fishery resource in East Asia (Zhao et al. 1998), the local extinction risk due to pollution, overfishing, and climate change may pose significant risks to fisheries sustainability and local economies. From this perspective, *E. sinensis* stocking programs initiated in China in the 1980s and in Korea in 1999 (Cha 2001; Wang et al. 2006) have the potential to facilitate population recovery when integrated with systematic genomic monitoring. However, the present dataset does not allow a direct assessment of the genetic impacts of past stocking efforts, and determining their contribution to contemporary population structure will require targeted sampling and temporal genomic comparisons in future studies. Overall, our study highlights the advantage of genomic resources for population and conservation genetics of *E. sinensis*, an economically important fishery species in East Asia.

## Supplementary Material

Table_S1-S5.xlsx

Figure S1.docx

## Data Availability

SLAF-seq data of 120 *Eriocheir sinensis* are available on NCBI as BioProject no. PRJNA1205783.
