# Supplementary material for: Genome-wide detection of fine-scale population stratification and long-distance dispersal of the Chinese mitten crab (Eriocheir sinensis)
Source: Anim Cells Syst (Seoul). 2026 Jan 31;30(1):131–45. doi: 10.1080/19768354.2026.2619207 (PMC12862865; doi:10.1080/19768354.2026.2619207)
Supplement: Figure S1.docx [file TACS_A_2619207_SM5284.docx]

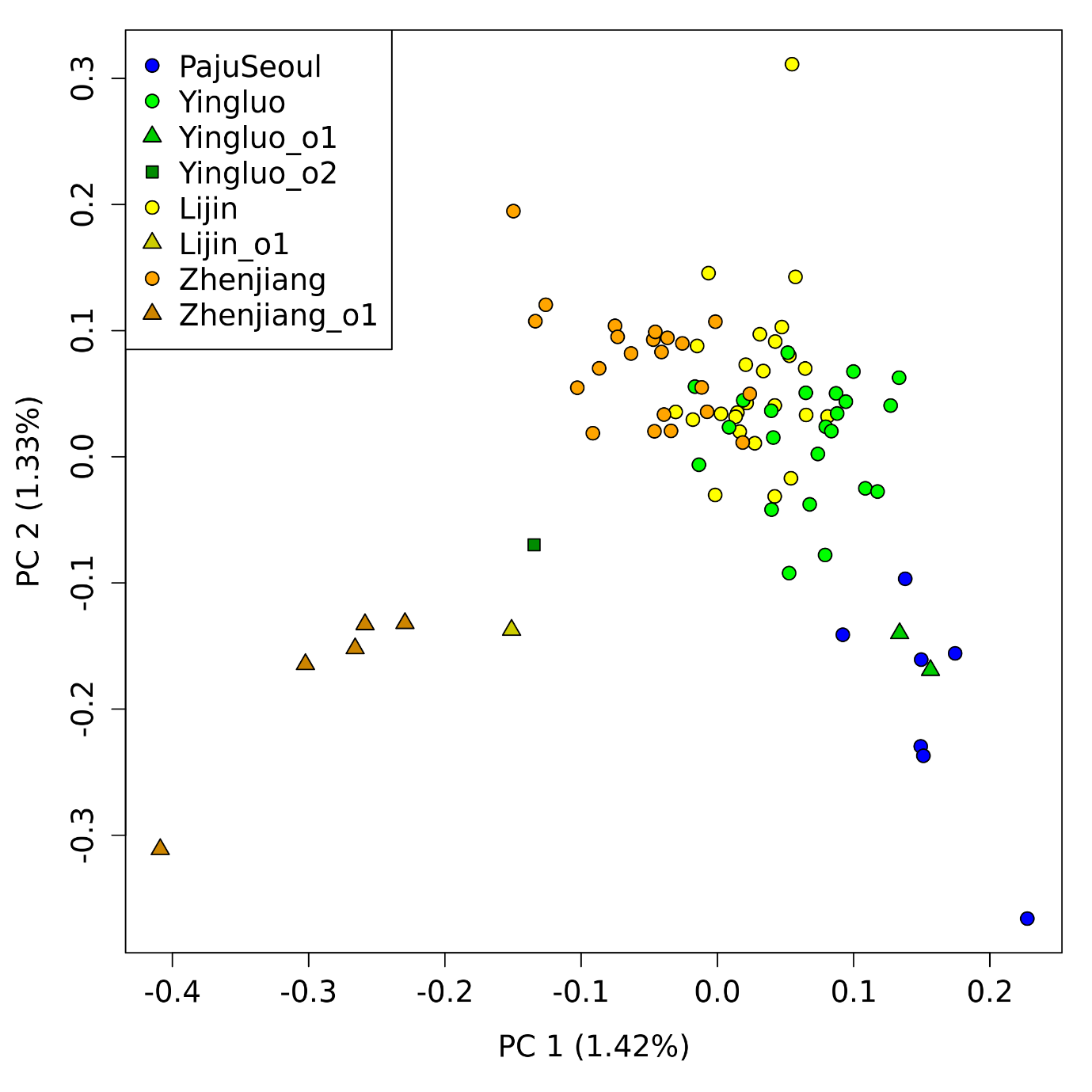


**Figure S1. PCA of 86 *E. sinensis* individuals excluding Seocheon and Wenzhou.** The top two PCs are plotted on the x- and y-axis, respectively. Numbers in parenthesis show the proportion of total variance in the genetic covariance matrix explained by each PC.
